# Dual-Motor Synchronization Control Design Based on Adaptive Neural Networks Considering Full-State Constraints and Partial Asymmetric Dead-Zone

**DOI:** 10.3390/s21134261

**Published:** 2021-06-22

**Authors:** Chunhong Jin, Mingjie Cai, Zhihao Xu

**Affiliations:** 1 School of Automation, Qingdao University, Qingdao 266071, China; 2019020549@qdu.edu.cn; 2 Shandong Key Laboratory of Industrial Control Technology, Qingdao 266071, China; 3 Guangdong Key Laboratory of Modern Control Technology, Institute of Intelligent Manufacturing, Guangdong Academy of Sciences, Guangzhou 510070, China; zh.xu@giim.ac.cn

**Keywords:** dual-motor servo systems, robot, neural networks, full-state constraints, time-varying barrier Lyapunov functions, command filtering backstepping

## Abstract

This paper proposes a command filtering backstepping (CFB) scheme with full-state constraints by leading into time-varying barrier Lyapunov functions (T-BLFs) for a dual-motor servo system with partial asymmetric dead-zone. Firstly, for the convenience of the controller design, the conventional partial asymmetric dead-zone model was replaced with a new smooth differentiable model owing to its non-smoothness. Secondly, neural networks (NNs) were utilized to approximate the nonlinearity that exists in the dead-zone model, improving the control performance. In addition, CFB was utilized to deal with the inherent computational explosion problem of the traditional backstepping method, and an error compensation mechanism was introduced to further reduce the filtering errors. Then, by applying the T-BLF to the CFB process, the states of the system never violated the prescribed constraints, and all signals in the dual-motor servo system were bounded. The tracking error and synchronization error could converge to a small desired neighborhood of the origin. In the end, the effectiveness of the proposed control scheme was verified through simulations.

## 1. Introduction

Over the past few decades, tracking control of motors have attracted considerable attention in the field of control theory and engineering [1,2,3,4,5]. Compared to dual-motor systems, it is difficult to satisfy the precision requirements of large inertia loads owing to the limited power of single motor systems. Therefore, dual-motor systems have recently been proposed and utilized in various applications [6,7,8,9,10] because of their advantages of high power, large inertia, and high-control performance. With the widespread usage of robots, especially industrial and agricultural robots, such as automatic assembly manipulator and high-precision automatic gantry hammock, large load and high-power application requirements are proposed. Thus, it is necessary to explore the control schemes of dual-motor systems for enabling robots or other control systems with large inertia to operate effectively. However, due to the complexity of dual-motor models, dealing with the nonlinearity and designing the control scheme for the systems brings great challenges.

It is universally acknowledged that backstepping technology is an effective tool for handling nonlinear dynamics. However, the controller design is complex when using traditional backstepping in dual-motor systems, and there also exists the problem of computational explosion. Thus, dynamic surface control (DSC) was introduced to overcome the drawback of “explosion of complexity”. In [11,12], the control methods for permanent magnet synchronous motors (PMSMs) were investigated. Dynamic surface control was utilized based on adaptive fuzzy logic (AFL) and NNs, respectively, which resolved the computational explosion problem, and the desired dynamic performance was achieved. The authors of [13] combined DSC with AFL in induction motors, guaranteeing that the closed-loop signals were bounded, and the tracking error converged to a small neighborhood of the origin. However, the problem of errors arose from the first-order filters, which was not considered in DSC, and which affected the control property. At the same time, they took no account of introducing an error compensation mechanism to obtain a better control performance for the controlled systems. Fortunately, a CFB approach was presented in [14,15,16,17] to solve the same problem, and the error compensation was also introduced to cope with the drawbacks of DSC. Thus, the computational burden of the design process was reduced, and the tracking error decreased. Fuzzy finite-time CFB was developed for position tracking control of induction motors with input saturation [15]. It guarantees the convergence of the tracking error in finite time and improves the dynamic performance of the control system. In [17], AFL via CFB was proposed for uncertain strict-feedback nonlinear systems with unknown non-symmetric dead-zone input signals. The aforementioned research was mainly focused on single motor systems or a class of nonlinear systems but is rarely applied to dual-motor systems. Although the above schemes have shown good control performances, they are not able to tackle the control problem when there are state or output constraints.

Obviously, severe performance degradation and safety problems or other problems can be caused by violation of these constraints. Constraints are widespread in most physical systems, and many methods have also been discussed to guarantee the stability and good control performance for various kinds of systems with the state or output constraints. Barrier Lyapunov functions (BLFs) have been proposed and used in the controller design of various systems to tackle the state constraints such as spacecraft [18], uncertain robot [19], hypersonic flight vehicles [20], and robotic manipulators [21]. For robot manipulators, a tracking controller considering the output error constraints was developed. It was guaranteed that the system could remain stable by using the bounded BLF when the output errors exceeded the constrained boundaries [21]. In this research, full-state constraints were handled using BLF, guaranteeing the uniform ultimate boundedness of the closed-loop system, and the constraints were never violated. Output constraints also became front-line research for researchers. The authors of [22,23] combined BLF with NNs to tackle the output constraint of a robotic manipulator with uncertainties and input dead-zone, respectively. In [24], the same methods were used to solve multiple output constraints for a fully actuated marine surface vessel. But in the abovementioned literature, constant constraints were adopted. In fact, in many practical situations, time-varying constraints are more realistic because of various changing factors. The integral barrier Lyapunov function (IBLF) was used in control design to guarantee the condition of output constraints for an uncertain 2-DOF helicopter system [25]. In [26], a control design without constraint and full-state constraint was considered, and IBLF was introduced to avoid the violation of the constraint. For a class of nonlinear strict-feedback systems with uncertain parameters, the asymmetric T-BLF was applied in each step of the backstepping approach to handle the full-state constraints in [27]. To sum up, driven by theoretical challenges and practical needs, the design scheme of constrained control has become a significant research topic. Thus, it is meaningful to consider the dual-motor servo system subject to full-state constraints.

Being universal approximators, fuzzy logic systems (FLSs) have been applied to identify the nonlinear terms in controller design [28,29]. For NNs, many outstanding results have also been given in various systems [30,31,32,33,34,35,36,37]. A new adaptive funnel controller based on the backstepping method was designed for the servo mechanism with friction in [31], and the nonlinear parts were approximated by NNs. In [33], the design of NNs using a broad learning framework was given. An adaptive neural controller was developed to ensure the tracking performance in the robot system with uncertainties [34]. In a robot learning system, NNs were used to deal with the effects of dynamic environments [36]. Similar to aforementioned literature, NNs were adopted to identify nonlinear functions in this paper.

However, to the authors’ best knowledge, there are few related studies regarding adaptive NNs based on CFB for a dual-motor servo system. On the other hand, the output and state constraints for various systems have become a hot research topic. This motivates the present study. Taking these factors into account, adaptive NNs based on CFB for the dual-motor servo system with full-state constraints was investigated in this paper.

In this paper, the nonlinearity was handled by CFB and adaptive NNs, which gives a systematic scheme to solve the nonlinear issues. The T-BLF was employed to tackle the full-state constraints of the dual-motor servo system. The simulation results prove that the whole control scheme improves the control performance of the system. Thus, the main contributions of this paper are summarized as follows:

(1) The mathematical model of the dual-motor servo system with the partial asymmetric dead-zone was re-established. The T-BLF was utilized to cope with the full-state constraints in the system so that the states were never transgressed;

(2) By using CFB, the issue of “explosion of complexity” that arises from the traditional backstepping in the dual-motor system was solved, and the error compensation mechanism introduced can effectively reduce the filtering errors to gain a smaller tracking error. It can be proved that the tracking error can converge to a small neighborhood of the origin;

(3) In dual-motor servo systems, adaptive NNs are used to approximate the nonlinear parts, improving the control precision of the system. By constructing suitable virtual controllers, the synchronization error eventually converges to a small neighborhood of the origin.

The remainder of this paper is organized as follows. The system descriptions and preliminaries in Section 2. The controller design in Section 3. The stability analysis is given in Section 4. Section 5 provides simulation results that illustrate the effectiveness of the proposed control scheme. Finally, the conclusions are drawn in Section 6.

## 2. System Descriptions and Preliminaries

**Figure 1:**
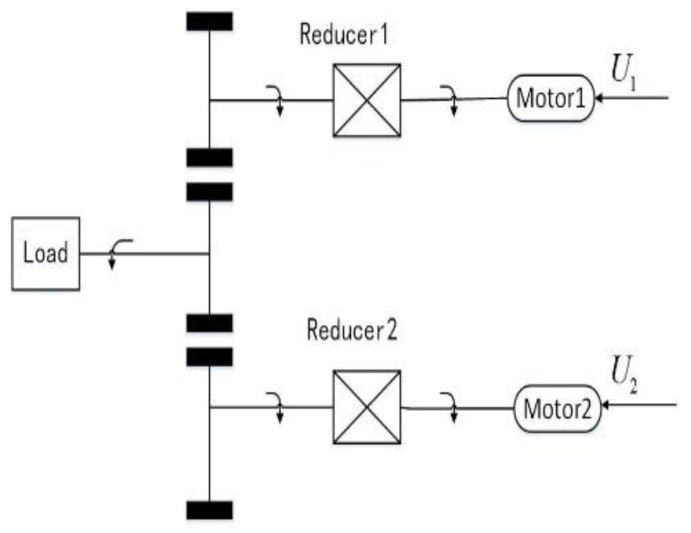
Structure diagram of the dual-motor synchronized driving servo system.

Consider the dynamic model of the dual-motor servo system with partial asymmetric dead-zone in the following form:

(1) $$\{\begin{array}{l} \begin{array}{c} {\dot{\theta }}_{L}(t)=\omega_{L}(t)\\ {\dot{\theta }}_{mj}(t)=\omega_{mj}(t) \end{array}\\ {\dot{\omega }}_{L}(t)=[Dead(\theta_{1})+Dead(\theta_{2})]/J_{L}-b_{L}\omega_{L}(t)/J_{L}\\ {\dot{\omega }}_{mj}(t)=[K_{tj}i_{j}(t)-Dead(\theta_{j})]/J_{mj}-b_{j}\omega_{mj}(t)/J_{mj}\\ {\dot{i}}_{j}(t)=[-R_{j}i_{j}(t)-K_{ej}\omega_{mj}(t)+U_{j}(t)]/L_{j}, \end{array}$$

where the subscript j(j=1,2) represents different groups of motor parameters, and $\theta_{mj},\omega_{mj},K_{tj},K_{ej},J_{mj},b_{j},i_{j},R_{j},L_{j},U_{j}$ are the angular position, angular velocity, electromagnetic torque constant, back electromotive force constant, inertia, viscous friction coefficients, current, resistance, inductance, and the control voltage of each motor, respectively. The angular position, angular velocity, inertia, and viscous friction coefficient of the load are converted to the motor shaft as $\theta_{L},\omega_{L},J_{L},b_{L}$. The structure diagram of the dual-motor synchronized driving servo system is shown in the following Figure 1.

$Dead(\theta_{j})$ is the transmission torque and is expressed as the partial asymmetric dead-zone:

(2) $$Dead(\theta_{j})=\left\{ \begin{array}{l} k(\theta_{j}-\partial_{r}),if\;\theta_{j}\geq \partial_{r}\\ 0,if\;\theta_{j}\in (-\partial_{l},\partial_{r})\\ k(\theta_{j}+\partial_{l}),if\;\theta_{j}\leq -\partial_{l}, \end{array}\right.$$

where $\theta_{j}=\theta_{mj}-\theta_{L}$, which is the relative displacement, k is the rigidity coefficient, $\partial_{r}$ and $\partial_{l}$ are break points satisfying $\partial_{r}>0$, $-\partial_{l}<0$. But the dead-zone model is non-smooth, resulting in collision and bringing great difficulty in the controller design. Thus, a new differentiable dead-zone model with non-symmetric break points is proposed as:

(3) $$T_{s}(\theta_{j})=k\theta_{j}+\frac{k}{2r}\ln(\frac{\cosh(r(\theta_{j}-\partial_{r}))}{\cosh(r(\theta_{j}+\partial_{l}))})+\frac{k}{2}(\partial_{l}-\partial_{r}),$$

in which r is called soft degree, a positive adjustable parameter. The meaning of other parameters is the same as in $Dead(\theta_{j})$. 

Let $\chi (\theta_{j})=Dead(\theta_{j})-T_{s}(\theta_{j})$ and combine (2) and (3), then we have:

(4) $$\chi (\theta_{j})=\left\{ \begin{array}{l} -k\partial_{r}-\frac{k\rho (\theta_{j})}{2r}-\frac{k}{2}(\partial_{l}-\partial_{r}),if\;\theta_{j}\geq \partial_{r}\\ -k\theta_{j}-\frac{k\rho (\theta_{j})}{2r}-\frac{k}{2}(\partial_{l}-\partial_{r}),if\;\theta_{j}\in (-\partial_{l},\partial_{r})\\ k\partial_{l}-\frac{k\rho (\theta_{j})}{2r}-\frac{k}{2}(\partial_{l}-\partial_{r}),if\;\theta_{j}\leq -\partial_{l}, \end{array}\right.$$

where $\rho (\theta_{j})=\ln(\frac{\cosh(r(\theta_{j}-\partial_{r}))}{\cosh(r(\theta_{j}+\partial_{l}))})$.

Owing to $d\rho /d\theta_{j}<0$, from (4) we can get:

(5) $$\left\{ \begin{array}{l} -k\partial_{r}-\frac{k}{2r}\ln(\frac{2}{e^{2r\partial_{r}}+e^{-2r\partial_{l}}})\leq \chi (\theta_{j})<0,if\;\theta_{j}\geq \partial_{r}\\ -k\partial_{r}-\frac{k}{2r}\ln(\frac{2}{e^{2r\partial_{r}}+e^{-2r\partial_{l}}})<\chi (\theta_{j})<k\partial_{l}+\frac{k}{2r}\ln(\frac{2}{e^{2r\partial_{l}}+e^{-2r\partial_{r}}}),if\;\theta_{j}\in (-\partial_{l},\partial_{r})\\ 0<\chi (\theta_{j})\leq k\partial_{l}+\frac{k}{2r}\ln(\frac{2}{e^{2r\partial_{l}}+e^{-2r\partial_{r}}}),if\;\theta_{j}\leq -\partial_{l}. \end{array}\right.$$

The Equation (5) shows that $-k\partial_{r}-\frac{k}{2r}\ln(\frac{2}{e^{2r\partial_{r}}+e^{-2r\partial_{l}}})\leq \chi (\theta_{j})\leq k\partial_{l}+\frac{k}{2r}\ln(\frac{2}{e^{2r\partial_{l}}+e^{-2r\partial_{r}}})$.

**Figure 2:**
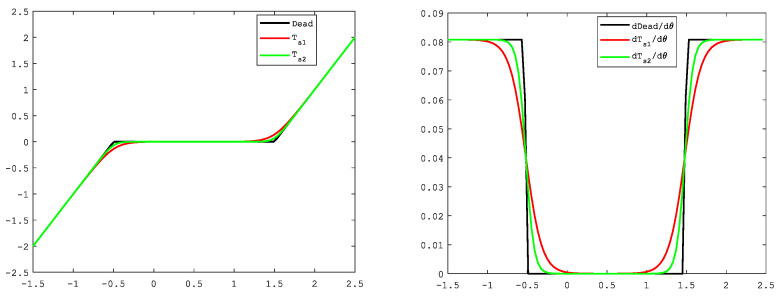
Dead-zone approximation: $k=2,\partial_{r}=1.5,\partial_{l}=0.5$.

Therefore, we can conclude that $-(k\ln2)/2r<\chi (\theta_{j})<(k\ln2)/2r$, and $\underset{r\to +\infty }{\lim}\left|\chi (\theta_{j})\right|=0$. It implies that the non-smooth property of the dead-zone nonlinearity can be smoothed to any arbitrary precision by an additional design parameter r in $T_{s}$. For instance, r=5 in $T_{s1}$ and r=10 in $T_{s2}$ as shown in Figure 2. The new dead-zone model greatly facilitates the controller design in practice.

The T-BLF candidate utilized in the control design process can be chosen as follows [38]:

(6) $$V_{i}=\frac{1}{2}\log(\frac{k_{b_{i}}^{2}(t)}{k_{b_{i}}^{2}(t)-v_{i}^{2}}),k_{b_{i}}(t)=(\iota_{i}-\psi_{i})e^{-\gamma_{i}t}+\psi_{i},i=1,2,3,4,5,$$

where $\iota_{i},\gamma_{i},\psi_{i}$ are positive adjustable parameters. $v_{i}$ will be defined in the following control design process. Define a compact set $\Omega_{v}:=\left\{\left|v_{i}\right|<k_{b_{i}}(t)\right\}$ and label $k_{v_{i}}=\frac{v_{i}}{k_{b_{i}}^{2}(t)-v_{i}^{2}}$.

All states of the dual-motor servo system are constrained in a compact set, for example, $\left|x_{i}\right|\leq k_{c_{i}}(t)$ with $k_{c_{i}}(t)>0$.

**Remark** **1.** *Owing to the existence of the constraints of control variables and state variables in many practical systems as well as the time-varying parameters, it is necessary to consider both the time-varying and constrained characteristics of the dual-motor servo system*.

Then, we let $a_{0}=k/J_{L},a_{1}=b_{L}/J_{L},\hbar_{1j}=K_{tj}/J_{mj},\hbar_{2j}=k/J_{mj},\hbar_{3j}=b_{j}/J_{mj},\hbar_{4j}=R_{j}/L_{j}$ and $\hbar_{5j}=K_{ej}/L_{j},\hbar_{6j}=1/L_{j}$. Define the state variables in the dual-motor servo system $x_{1}=\theta_{L},x_{2}=\omega_{L},x_{3j}=\theta_{mj},x_{4j}=\omega_{mj},x_{5j}=i_{j}$.

Therefore, the state equations can be rewritten as:

(7) $$\left\{ \begin{array}{l} {\dot{x}}_{1}=x_{2}\\ {\dot{x}}_{2}=a_{0}x_{3}-2a_{0}x_{1}-a_{1}x_{2}+\Phi_{2}+\Phi_{3}\\ {\dot{x}}_{3j}=x_{4j}\\ {\dot{x}}_{4j}=\hbar_{1j}x_{5j}-\hbar_{2j}(x_{3j}-x_{1})+\Phi_{4j}-\Phi_{5j}-\hbar_{3j}x_{4j}\\ {\dot{x}}_{5j}=-\hbar_{4j}x_{5j}-\hbar_{5j}x_{4j}+\hbar_{6j}U_{j}\\ y=x_{1}, \end{array}\right.$$

where $\Phi_{2}=[\frac{k}{2r}(\rho (\theta_{1})+\rho (\theta_{2}))+k(\partial_{l}-\partial_{r})]/J_{L},\Phi_{3}=(\chi (\theta_{1})+\chi (\theta_{2}))/J_{L},\Phi_{5j}=\chi (\theta_{j})/J_{mj}$ and $\Phi_{4j}=[-\frac{k}{2r}\rho (\theta_{j})-\frac{k}{2}(\partial_{l}-\partial_{r})]/J_{mj}$.

For the convenience of control design, the following lemmas are given.

**Lemma 1** **([38]).** *For any constant*$k_{b}>0$, *and any*z∈R*satisfying*$\left|z\right|<k_{b}$, *we have:*

(8) $$\log(\frac{k_{b}^{2}}{k_{b}^{2}-z^{2}})<\frac{z^{2}}{k_{b}^{2}-z^{2}}.$$

**Lemma 2** **([39]).** *The NNs are employed to approximate a continuous function*f(x). *The approximation of function*f(x)*over a compact domain*Ω*is defined as:*

(9) $$f(x)=W^{*}{}^{T}S(x)+\varsigma (x),\forall x\in \Omega .$$

$S(x)={\left[s_{1}(x),s_{2}(x),\cdots ,s_{l}(x)\right]}^{T}$*is the basis function vector, and*l>0*denotes the node number of NNs.*ς(x)*is the approximation error.*$W^{*}$*is the ideal value of the NNs’ weight that minimizes the approximation error*ς(x)*. Thus, we have:*$W^{*}=\arg\underset{W\in R^{L}}{\min}\left\{\underset{x\in \Omega }{\sup}\left|f(x)-W^{*}{}^{T}S(x)\right|\right\}.$*A Gaussian function is usually chosen as the basis function*$s_{i}(x)$*, that is:*$s_{i}(x)=\exp[-\frac{{\left(x-\varpi_{i}\right)}^{T}(x-\varpi_{i})}{\eta_{i}^{2}}],i=1,2,\cdots ,l,$*in which*$\varpi_{i}=[\varpi_{i1},\varpi_{i2},\cdots ,\varpi_{in}]$*is the center of the basis function and*$\eta_{i}$*is the width. Because the ideal NNs weight,*$W^{*}$, *is unknown, we can only use the estimation value*$\hat{W}$*of*$W^{*}$*in the control design, which can be updated online via adaptive laws.*

**Assumption** **1.** *For any*x∈Ω, *the approximation error satisfies*$\left|\varsigma (x)\right|\leq \varepsilon_{M}$, *where*$\varepsilon_{M}>0$*is an unknown bound*.

**Remark** **2.** *NNs have been widely utilized in the modeling and control of nonlinear systems with unknown dynamics by using their approximations and learning abilities, so NNs were employed to approximate nonlinearity to obtain good control performance in this paper*.

**Lemma 3** **([40]).** *Command filter was defined as:*

(10) $$\left\{ \begin{array}{l} {\dot{\varphi }}_{1}=\omega_{n}\varphi_{2}\\ {\dot{\varphi }}_{2}=-2\zeta \omega_{n}\varphi_{2}-\omega_{n}(\varphi_{1}-\alpha ). \end{array}\right.$$

*If the input signal*α*satisfies*$\left|\dot{\alpha }\right|\leq \lambda_{1}$*and*$\left|\ddot{\alpha }\right|\leq \lambda_{2}$*for all*t≥0*, where*$\lambda_{1}$*and*$\lambda_{2}$*are positive constants*, $\varphi_{1}(0)=\alpha (0)$*and*$\varphi_{2}(0)=0$, *then for any*β>0, *there exist*$\omega_{n}>0$*and*ζ∈(0,1], *such that*$\left|\varphi_{1}-\alpha \right|\leq \beta$, $\left|{\dot{\varphi }}_{1}\right|$, $\left|{\ddot{\varphi }}_{1}\right|$, *and*$\left|{\dddot{\varphi }}_{1}\right|$*are bounded*.

**Assumption** **2.** $y_{d}$*and its first derivative*${\dot{y}}_{d}$*are known, bounded and smooth with*$\left|y_{d}\right|\leq k_{c_{1}}(t)$.

The control objective was to design a smooth CFB controller with an appropriate selection of control parameters such that (1) all the closed-loop signals of the dual-motor servo system with a partial asymmetric dead-zone were bounded and the state constraints were never violated and (2) the output, $x_{1}$, followed the specified desired trajectory, $y_{d}$, so that the tracking error was uniformly ultimately bounded with practical accuracy. Meanwhile, the speed synchronization error converged to a small neighborhood of the origin.

## 3. Controller Design of Command Filtering Backstepping with Full-State Constraints

In this section, for the purpose of alleviating the high complexity, an adaptive NN controller based on CFB is presented for the dual-motor servo system (1) by employing T-BLF. The development procedure was composed of five steps, and the detailed process is elaborated as follows.

Owing to the error compensation mechanism utilized in this paper, the compensated tracking error was designed as $v_{i}=z_{i}-\xi_{i},i=1,2,3,4,5$. $z_{i}$, as the tracking error, is given later, and $\xi_{i}$ is the error compensation signal as:

(11) $$\left\{ \begin{array}{l} {\dot{\xi }}_{1}=-k_{1}\xi_{1}+\xi_{2}+(x_{2,c}-\alpha_{1})\\ {\dot{\xi }}_{2}=-k_{2}\xi_{2}+\xi_{3}+(x_{3,c}-\alpha_{2})\\ {\dot{\xi }}_{3}=-k_{3}\xi_{3}+\xi_{4}+(x_{4,c}-\alpha_{3})\\ {\dot{\xi }}_{4}=-k_{4}\xi_{4}+\xi_{5}+(x_{51,c}-\alpha_{41}+x_{52,c}-\alpha_{42})\\ {\dot{\xi }}_{5}=-k_{5}\xi_{5}, \end{array}\right.$$

where $\xi_{i}(0)=0,i=1,2,3,4,5$. The compensation signal, $\xi_{i}$, is bounded and denoted as $\underset{t\to \infty }{\lim}\left|\xi_{i}\right|\leq \sqrt{2m_{0}/n_{0}}$, the $m_{0},n_{0}$ is defined in a later proof.

In (11), $(x_{i,c}-\alpha_{i-1}),i=2,3,4$ and $\left(x_{51,c}-\alpha_{41}+x_{52,c}-\alpha_{42}\right)$ are the filtering errors, which may bring difficulty in obtaining a satisfactory control performance. $x_{i,c},x_{51,c}$ and $x_{52,c}$ are the output signals of the command filtering, while the virtual controllers $\alpha_{i-1},\alpha_{41}$ and $\alpha_{42}$ go through the filter. The virtual controllers are defined in the process of controller design.

**Step1:** According to the control objective of the system (1) and Equation (7), the first tracking error was defined as $z_{1}=x_{1}-y_{d}$. The time derivative of $z_{1}$ is ${\dot{z}}_{1}={\dot{x}}_{1}-{\dot{y}}_{d}=x_{2}-{\dot{y}}_{d}$, where $y_{d}$ is the reference signal.

In order to make the system states constrained, the first T-BLF candidate was chosen as:

(12) $$V_{1}=\frac{1}{2}\log(\frac{k_{b_{1}}^{2}(t)}{k_{b_{1}}^{2}(t)-v_{1}^{2}}).$$

Then, the time derivative of $V_{1}$ can be deduced by:

(13) $$\begin{array}{ll} {\dot{V}}_{1} & =\frac{v_{1}{\dot{v}}_{1}-v_{1}^{2}[{\dot{k}}_{b_{1}}(t)/k_{b_{1}}(t)]}{k_{b_{1}}^{2}(t)-v_{1}^{2}}\\ & =k_{v_{1}}[{\dot{v}}_{1}-v_{1}({\dot{k}}_{b_{1}}(t)/k_{b_{1}}(t))]\\ & =k_{v_{1}}[{\dot{x}}_{1}-{\dot{y}}_{d}-{\dot{\xi }}_{1}-v_{1}({\dot{k}}_{b_{1}}(t)/k_{b_{1}}(t))]\\ & =k_{v_{1}}[x_{2}-x_{2,c}+x_{2,c}-\alpha_{1}+\alpha_{1}-{\dot{y}}_{d}-{\dot{\xi }}_{1}-v_{1}({\dot{k}}_{b_{1}}(t)/k_{b_{1}}(t))]. \end{array}$$

The first virtual controller is constructed as:

(14) $$\alpha_{1}=-k_{1}z_{1}+{\dot{y}}_{d}+v_{1}({\dot{k}}_{b_{1}}(t)/k_{b_{1}}(t)),$$

where $k_{1}$ is a positive adjustable parameter.

By substituting (11) and (14) to (13), we have:

(15) $$\begin{array}{ll} {\dot{V}}_{1} & =k_{v_{1}}(z_{2}-k_{1}z_{1}+k_{1}\xi_{1}-\xi_{2})\\ & \leq k_{v_{1}}v_{2}-k_{v_{1}}k_{1}v_{1}. \end{array}$$

**Step2:** The second tracking error was $z_{2}=x_{2}-x_{2,c}$; thus, the time derivative of $z_{2}$ is ${\dot{z}}_{2}={\dot{x}}_{2}-{\dot{x}}_{2,c}=a_{0}x_{3}-2a_{0}x_{1}-a_{1}x_{2}+\Phi_{2}+\Phi_{3}-{\dot{x}}_{2,c}$.

The T-BLF candidate was defined as:

(16) $$V_{2}=V_{1}+\frac{1}{2}\log(\frac{k_{b_{2}}^{2}(t)}{k_{b_{2}}^{2}(t)-v_{2}^{2}}).$$

Then, the time derivative of $V_{2}$ is:

(17) $$\begin{array}{ll} {\dot{V}}_{2} & ={\dot{V}}_{1}+\frac{v_{2}{\dot{v}}_{2}-v_{2}^{2}[{\dot{k}}_{b_{2}}(t)/k_{b_{2}}(t)]}{k_{b_{2}}^{2}(t)-v_{2}^{2}}\\ & ={\dot{V}}_{1}+k_{v_{2}}[{\dot{x}}_{2}-{\dot{x}}_{2,c}-{\dot{\xi }}_{2}-v_{2}({\dot{k}}_{b_{2}}(t)/k_{b_{2}}(t))]\\ & ={\dot{V}}_{1}+k_{v_{2}}[a_{0}x_{3}-x_{3,c}+x_{3,c}-\alpha_{2}+\alpha_{2}-2a_{0}x_{1}-a_{1}x_{2}+\Phi_{2}+\Phi_{3}-{\dot{x}}_{2,c}-{\dot{\xi }}_{2}\\ & -v_{2}({\dot{k}}_{b_{2}}(t)/k_{b_{2}}(t))]. \end{array}$$

Select the second virtual controller:

(18) $$\begin{array}{ll} \alpha_{2} & =-k_{2}z_{2}+{\dot{x}}_{2,c}+2a_{0}x_{1}+a_{1}x_{2}+{\hat{W}}_{2}^{T}S_{2}-k_{v_{1}}(k_{b_{2}}^{2}-v_{2}^{2})-k_{v_{2}}\\ & +v_{2}({\dot{k}}_{b_{2}}(t)/k_{b_{2}}(t)), \end{array}$$

where $k_{2}$ is a positive adjustable parameter, and ${\hat{W}}_{2}$ is the estimation value of $W_{2}^{*}$.

By substituting (11) and (18) to (17) yields:

(19) $$\begin{array}{ll} {\dot{V}}_{2} & ={\dot{V}}_{1}+k_{v_{2}}(z_{3}-k_{2}z_{2}+{\hat{W}}_{2}^{T}S_{2}-k_{v_{1}}(k_{b_{2}}^{2}-v_{2}^{2})-k_{v_{2}}+\Phi_{2}+\Phi_{3}+k_{2}\xi_{2}-\xi_{3})\\ & ={\dot{V}}_{1}+k_{v_{2}}(v_{3}-k_{2}v_{2}+({\tilde{W}}_{2}^{T}S_{2}-\varsigma_{2})-k_{v_{1}}(k_{b_{2}}^{2}-v_{2}^{2})-k_{v_{2}}+\Phi_{3})\\ & =-\sum_{i=1}^{2}k_{v_{i}}k_{i}v_{i}+k_{v_{2}}v_{3}+k_{v_{2}}{\tilde{W}}_{2}^{T}S_{2}-k_{v_{2}}\varsigma_{2}+k_{v_{2}}\Phi_{3}-k_{v_{2}}^{2}, \end{array}$$

in which $\Phi_{2}=f_{2}(x)=W_{2}^{*T}S_{2}(x)+\varsigma_{2}(x)$. 

We have $\Phi_{3}=\left(\chi (\theta_{1})+\chi (\theta_{2})\right)/J_{L}$ and $-(k\ln2)/2r<\chi (\theta_{j})<(k\ln2)/2r$, so $\Phi_{3}<H_{2}$. In addition, $k_{v_{2}}\Phi_{3}$ satisfies $k_{v_{2}}\Phi_{3}\leq \frac{1}{2}k_{v_{2}}^{2}+\frac{1}{2}H_{2}^{2}$ according to the Young’s inequality. Similarly, from Assumption 1, we can obtain $-k_{v_{2}}\varsigma_{2}\leq \frac{1}{2}k_{v_{2}}^{2}+\frac{1}{2}\varepsilon_{2}^{2}$, since $\varsigma_{2}\leq \varepsilon_{2}$. $H_{2}$ is a positive parameter, $\varepsilon_{2}$ is an unknown bound, and $\varsigma_{2}$ is the approximation error in this procedure. 

Therefore, we have the final result of taking the time derivative of $V_{2}$:

(20) $$\begin{array}{ll} {\dot{V}}_{2} & \leq -\sum_{i=1}^{2}k_{v_{i}}k_{i}v_{i}+k_{v_{2}}v_{3}+k_{v_{2}}{\tilde{W}}_{2}^{T}S_{2}+\frac{1}{2}k_{v_{2}}^{2}+\frac{1}{2}\varepsilon_{2}^{2}+\frac{1}{2}k_{v_{2}}^{2}+\frac{1}{2}H_{2}^{2}-k_{v_{2}}^{2}\\ & =-\sum_{i=1}^{2}k_{v_{i}}k_{i}v_{i}+k_{v_{2}}v_{3}+k_{v_{2}}{\tilde{W}}_{2}^{T}S_{2}+\frac{1}{2}\varepsilon_{2}^{2}+\frac{1}{2}H_{2}^{2}. \end{array}$$

**Step3:** Design the third tracking error $z_{3}=a_{0}x_{3}-x_{3,c}$, and its time derivative is ${\dot{z}}_{3}=a_{0}{\dot{x}}_{3}-{\dot{x}}_{3,c}=a_{0}x_{4}-{\dot{x}}_{3,c}$.

The T-BLF candidate in this step was chosen as:

(21) $$V_{3}=V_{2}+\frac{1}{2}\log(\frac{k_{b_{3}}^{2}(t)}{k_{b_{3}}^{2}(t)-v_{3}^{2}}).$$

Analogously, differentiating $V_{3}$ with respect to time, we obtain:

(22) $$\begin{array}{ll} {\dot{V}}_{3} & ={\dot{V}}_{2}+\frac{v_{3}{\dot{v}}_{3}-v_{3}^{2}[{\dot{k}}_{b_{3}}(t)/k_{b_{3}}(t)]}{k_{b_{3}}^{2}(t)-v_{3}^{2}}\\ & ={\dot{V}}_{2}+k_{v_{3}}[a_{0}{\dot{x}}_{3}-{\dot{x}}_{3,c}-{\dot{\xi }}_{3}-v_{3}({\dot{k}}_{b_{3}}(t)/k_{b_{3}}(t))]\\ & ={\dot{V}}_{2}+k_{v_{3}}[a_{0}x_{4}-x_{4,c}+x_{4,c}-\alpha_{3}+\alpha_{3}-{\dot{x}}_{3,c}-{\dot{\xi }}_{3}-v_{3}({\dot{k}}_{b_{3}}(t)/k_{b_{3}}(t))]. \end{array}$$

The virtual controller is designed as:

(23) $$\alpha_{3}=-k_{3}z_{3}+{\dot{x}}_{3,c}-k_{v_{2}}(k_{b_{3}}^{2}-v_{3}^{2})+v_{3}({\dot{k}}_{b_{3}}(t)/k_{b_{3}}(t)),$$

where $k_{3}$ is a positive adjustable parameter.

By introducing (11) and (23) to (22) yields:

(24) $$\begin{array}{ll} {\dot{V}}_{3} & ={\dot{V}}_{2}+k_{v_{3}}[z_{4}-k_{3}z_{3}-k_{v_{2}}(k_{b_{3}}^{2}-v_{3}^{2})+k_{3}\xi_{3}-\xi_{4}]\\ & \leq -\sum_{i=1}^{3}k_{v_{i}}k_{i}v_{i}+k_{v_{3}}v_{4}+k_{v_{2}}{\tilde{W}}_{2}^{T}S_{2}+\frac{1}{2}\varepsilon_{2}^{2}+\frac{1}{2}H_{2}^{2}. \end{array}$$

**Step4:** The tracking error in the fourth subsystem was $z_{4j}=a_{0}x_{4j}-\frac{1}{2}x_{4,c},j=1,2$. From (7), we can obtain ${\dot{z}}_{4j}=a_{0}{\dot{x}}_{4j}-\frac{1}{2}{\dot{x}}_{4,c}=a_{0}(\hbar_{1j}x_{5j}-\hbar_{2j}(x_{3j}-x_{1})+\Phi_{4j}-\Phi_{5j}-\hbar_{3j}x_{4j})-\frac{1}{2}{\dot{x}}_{4,c}$. Define $z_{4}=z_{41}+z_{42},z_{s}=z_{42}-z_{41}$. $z_{s}$ is the speed synchronization error between two motors, which was used later.

The T-BLF candidate can be selected as:

(25) $$V_{4}=V_{3}+\frac{1}{2}\log(\frac{k_{b_{4}}^{2}(t)}{k_{b_{4}}^{2}(t)-v_{4}^{2}}).$$

Then, the time derivative of $V_{4}$ can be deduced by:

(26) $$\begin{array}{ll} {\dot{V}}_{4} & ={\dot{V}}_{3}+\frac{v_{4}{\dot{v}}_{4}-v_{4}^{2}[{\dot{k}}_{b_{4}}(t)/k_{b_{4}}(t)]}{k_{b_{4}}^{2}(t)-v_{4}^{2}}\\ & ={\dot{V}}_{3}+k_{v_{4}}[a_{0}{\dot{x}}_{4}-{\dot{x}}_{4,c}-{\dot{\xi }}_{4}-v_{4}({\dot{k}}_{b_{4}}(t)/k_{b_{4}}(t))]\\ & ={\dot{V}}_{3}+k_{v_{4}}[a_{0}(\hbar_{11}x_{51}-\hbar_{21}(x_{31}-x_{1})+\Phi_{41}-\Phi_{51}-\hbar_{31}x_{41}+\hbar_{12}x_{52}-\\ & \hbar_{22}(x_{32}-x_{1})+\Phi_{42}-\Phi_{52}-\hbar_{32}x_{42})-{\dot{x}}_{4,c}-{\dot{\xi }}_{4}-v_{4}({\dot{k}}_{b_{4}}(t)/k_{b_{4}}(t))]\\ & ={\dot{V}}_{3}+k_{v_{4}}[a_{0}\hbar_{11}x_{51}-x_{51,c}+a_{0}\hbar_{12}x_{52}-x_{52,c}+(x_{51,c}-\alpha_{41}+x_{52,c}-\alpha_{42})+\alpha_{41}\\ & +\alpha_{42}+a_{0}(-\hbar_{21}x_{31}+\hbar_{21}x_{1}-\hbar_{31}x_{41}-\hbar_{22}x_{32}+\hbar_{22}x_{1}-\hbar_{32}x_{42})+a_{0}(\Phi_{41}+\Phi_{42})\\ & -a_{0}(\Phi_{51}+\Phi_{52})-{\dot{x}}_{4,c}-{\dot{\xi }}_{4}-v_{4}({\dot{k}}_{b_{4}}(t)/k_{b_{4}}(t))]. \end{array}$$

Design the virtual controller:

(27) $$\begin{array}{ll} \alpha_{4j} & =-k_{4}z_{4j}+\frac{1}{2}{\dot{x}}_{4,c}+a_{0}(\hbar_{2j}(x_{3j}-x_{1})+\hbar_{3j}x_{4j})+{\hat{W}}_{4j}^{T}S_{4j}\\ & -\frac{1}{2}k_{v_{3}}(k_{b_{4}}^{2}-v_{4}^{2})-k_{v_{4}}+\frac{1}{2}v_{4}({\dot{k}}_{b_{4}}(t)/k_{b_{4}}(t))+{\left(-1\right)}^{j+1}k_{S}z_{s}, \end{array}$$

where $k_{4}$ and $k_{S}$ are positive adjustable parameters, and ${\hat{W}}_{4j}$ is the estimation value of $W_{4j}^{*}$.

**Remark** **3.** ${\left(-1\right)}^{j+1}k_{S}z_{s}$*in*$\alpha_{4j}$*is the synchronization feedback signal, which is designed to decrease the synchronization error between two motors*.

By substituting (11) and (27) to (26), we have:

(28) $$\begin{array}{ll} {\dot{V}}_{4} & \leq -\sum_{i=1}^{4}k_{v_{i}}k_{i}v_{i}+k_{v_{4}}v_{5}+k_{v_{2}}{\tilde{W}}_{2}^{T}S_{2}+\frac{1}{2}\varepsilon_{2}^{2}+\frac{1}{2}H_{2}^{2}+k_{v_{4}}{\tilde{W}}_{41}^{T}S_{41}+k_{v_{4}}{\tilde{W}}_{42}^{T}S_{42}\\ & -k_{v_{4}}\varsigma_{41}-k_{v_{4}}\varsigma_{42}-a_{0}k_{v_{4}}\Phi_{51}-a_{0}k_{v_{4}}\Phi_{52}-2k_{v_{4}}^{2}. \end{array}$$

Similar to step2, there exists $\Phi_{4j}=f_{4j}(x)=W_{4j}^{*T}S_{4j}(x)+\varsigma_{4j}(x)$ in this step. We can get $-k_{v_{4}}\varsigma_{41}\leq \frac{1}{2}\varepsilon_{41}^{2}+\frac{1}{2}k_{v_{4}}^{2}$ and $-k_{v_{4}}\varsigma_{42}\leq \frac{1}{2}\varepsilon_{42}^{2}+\frac{1}{2}k_{v_{4}}^{2}$ by combining the Young’s inequality and Assumption 1, in which $\varsigma_{41}<\varepsilon_{41},\varsigma_{42}<\varepsilon_{42}$ are satisfied. $\varepsilon_{41},\varepsilon_{42}$ are unknown bound, and $\varsigma_{41},\varsigma_{42}$ are the approximation errors. We have known $\Phi_{5j}=\chi (\theta_{j})/J_{mj}$ and $-(k\ln2)/2r<\chi (\theta_{j})<(k\ln2)/2r$, thus $-a_{0}\Phi_{51}<H_{41}$ and $-a_{0}\Phi_{52}<\frac{1}{2}H_{42}$ are obtained. $H_{41}$ and $H_{42}$ are positive parameters. In the end, we have $-a_{0}k_{v_{4}}\Phi_{51}\leq \frac{1}{2}H_{41}^{2}+\frac{1}{2}k_{v_{4}}^{2}$ and $-a_{0}k_{v_{4}}\Phi_{52}\leq \frac{1}{2}H_{42}^{2}+\frac{1}{2}k_{v_{4}}^{2}$ in accordance with the Young’s inequality.

Therefore, we obtain:

(29) $$\begin{array}{ll} {\dot{V}}_{4} & \leq -\sum_{i=1}^{4}k_{v_{i}}k_{i}v_{i}+k_{v_{4}}v_{5}+k_{v_{2}}{\tilde{W}}_{2}^{T}S_{2}+k_{v_{4}}{\tilde{W}}_{41}^{T}S_{41}+k_{v_{4}}{\tilde{W}}_{42}^{T}S_{42}+\frac{1}{2}\varepsilon_{2}^{2}+\frac{1}{2}H_{2}^{2}+\\ & \frac{1}{2}\varepsilon_{41}^{2}+\frac{1}{2}H_{41}^{2}+\frac{1}{2}\varepsilon_{42}^{2}+\frac{1}{2}H_{42}^{2}. \end{array}$$

**Step5:** The tracking error in this subsystem was designed as $z_{5j}=a_{0}\hbar_{1j}x_{5j}-x_{5j,c},j=1,2$. Then, its time derivative is ${\dot{z}}_{5j}=a_{0}\hbar_{1j}{\dot{x}}_{5j}-{\dot{x}}_{5j,c}=a_{0}\hbar_{1j}(-\hbar_{4j}x_{5j}-\hbar_{5j}x_{4j}+\hbar_{6j}u_{j})-{\dot{x}}_{5j,c}$. Define $z_{5}=z_{51}+z_{52}$ and $z_{T}=z_{52}-z_{51}$. $z_{T}$ is the torque synchronization error between two motors, which is also used later.

The T-BLF candidate is defined as:

(30) $$V_{5}=V_{4}+\frac{1}{2}\log(\frac{k_{b_{5}}^{2}(t)}{k_{b_{5}}^{2}(t)-v_{5}^{2}}).$$

Then, we have the time derivative of $V_{5}$:

(31) $$\begin{array}{ll} {\dot{V}}_{5} & ={\dot{V}}_{4}+\frac{v_{5}{\dot{v}}_{5}-v_{5}^{2}[{\dot{k}}_{b_{5}}(t)/k_{b_{5}}(t)]}{k_{b_{5}}^{2}(t)-v_{5}^{2}}\\ & ={\dot{V}}_{4}+k_{v_{5}}[a_{0}\hbar_{11}{\dot{x}}_{51}-{\dot{x}}_{51,c}+a_{0}\hbar_{12}{\dot{x}}_{52}-{\dot{x}}_{52,c}-{\dot{\xi }}_{5}-v_{5}({\dot{k}}_{b_{5}}(t)/k_{b_{5}}(t))]\\ & ={\dot{V}}_{4}+k_{v_{5}}[a_{0}\hbar_{11}(-\hbar_{41}x_{51}-\hbar_{51}x_{41}+\hbar_{61}u_{1})-{\dot{x}}_{51,c}+a_{0}\hbar_{12}(-\hbar_{42}x_{52}\\ & -\hbar_{52}x_{42}+\hbar_{62}u_{2})-{\dot{x}}_{52,c}-{\dot{\xi }}_{5}-v_{5}({\dot{k}}_{b_{5}}(t)/k_{b_{5}}(t))]. \end{array}$$

Construct the actual control signal as:

(32) $$\begin{array}{ll} U_{j} & =\frac{1}{a_{0}\hbar_{1j}\hbar_{6j}}[-k_{5}z_{5j}+{\dot{x}}_{5j,c}+a_{0}\hbar_{1j}(\hbar_{4j}x_{5j}+\hbar_{5j}x_{4j})-\frac{1}{2}k_{v_{4}}(k_{b_{5}}^{2}-v_{5}^{2})\\ & +\frac{1}{2}v_{5}({\dot{k}}_{b_{5}}(t)/k_{b_{5}}(t))]+{\left(-1\right)}^{j+1}\frac{1}{\hbar_{1j}\hbar_{6j}}[k_{T}z_{T}+\frac{1}{2a_{0}}z_{s}], \end{array}$$

in which $k_{5}$ and $k_{T}$ are positive adjustable parameters.

**Remark** **4.** *In*$U_{j}$, ${\left(-1\right)}^{j+1}\frac{1}{\hbar_{1j}\hbar_{6j}}[k_{T}z_{T}+\frac{1}{2a_{0}}z_{s}]$*is also the synchronization feedback signal. In order to improve the control accuracy and avoid unnecessary energy consumption in the dual-motor servo system, synchronization feedback signals are designed*.

Finally, substituting (11) and (32) into (31) yields:

(33) $$\begin{array}{ll} {\dot{V}}_{5} & \leq -\sum_{i=1}^{5}k_{v_{i}}k_{i}v_{i}+k_{v_{2}}{\tilde{W}}_{2}^{T}S_{2}+k_{v_{4}}{\tilde{W}}_{41}^{T}S_{41}+k_{v_{4}}{\tilde{W}}_{42}^{T}S_{42}+\frac{1}{2}\varepsilon_{2}^{2}+\frac{1}{2}H_{2}^{2}+\\ & \frac{1}{2}\varepsilon_{41}^{2}+\frac{1}{2}H_{41}^{2}+\frac{1}{2}\varepsilon_{42}^{2}+\frac{1}{2}H_{42}^{2}. \end{array}$$

## 4. Stability Analysis

**Theorem** **1.** *Considering the dual-motor servo system (1) satisfying Assumptions 1 and 2, the virtual controllers (14), (18), (23), (27) and actual controller (32), along with the adaptive laws (36) and compensating signals (11) are constructed. If the control design parameters are all appropriately selected, it can be ensured that tracking error and synchronization error converge to a small neighborhood of the origin. In addition, all the signals in this closed-loop system are bounded and the state constraints are never violated*.

**Proof** **of** **Theorem** **1.** The total Lyapunov function for the dual-motor servo system can be written as

(34) $$V=V_{5}+\frac{1}{2}{\tilde{W}}_{2}{}^{T}\Gamma_{2}^{-1}{\tilde{W}}_{2}+\frac{1}{2}{\tilde{W}}_{41}{}^{T}\Gamma_{41}^{-1}{\tilde{W}}_{41}+\frac{1}{2}{\tilde{W}}_{42}{}^{T}\Gamma_{42}^{-1}{\tilde{W}}_{42}+\frac{1}{2}z_{s}^{2}+\frac{1}{2}z_{T}^{2}.$$

**Remark** **5.** *There exist synchronization error and torque error in the studied system, so they are added to the total Lyapunov function, guaranteeing the convergence of these errors*.

Combining with (33), the derivative of V with respect to time can be deduced by:

(35) $$\begin{array}{ll} \dot{V} & ={\dot{V}}_{5}+{\tilde{W}}_{2}{}^{T}\Gamma_{2}^{-1}{\dot{\hat{W}}}_{2}+{\tilde{W}}_{41}{}^{T}\Gamma_{41}^{-1}{\dot{\hat{W}}}_{41}+{\tilde{W}}_{42}{}^{T}\Gamma_{42}^{-1}{\dot{\hat{W}}}_{42}+z_{s}{\dot{z}}_{s}+z_{T}{\dot{z}}_{T}\\ & \leq -\sum_{i=1}^{5}k_{v_{i}}k_{i}v_{i}+{\tilde{W}}_{2}^{T}(k_{v_{2}}S_{2}+\Gamma_{2}^{-1}{\dot{\hat{W}}}_{2})+{\tilde{W}}_{41}^{T}(k_{v_{4}}S_{41}+\Gamma_{41}^{-1}{\dot{\hat{W}}}_{41})+\\ & {\tilde{W}}_{42}^{T}(k_{v_{4}}S_{42}+\Gamma_{42}^{-1}{\dot{\hat{W}}}_{42})+z_{s}{\dot{z}}_{s}+z_{T}{\dot{z}}_{T}+\frac{1}{2}\varepsilon_{2}^{2}+\frac{1}{2}H_{2}^{2}+\frac{1}{2}\varepsilon_{41}^{2}+\\ & \frac{1}{2}H_{41}^{2}+\frac{1}{2}\varepsilon_{42}^{2}+\frac{1}{2}H_{42}^{2}. \end{array}$$

According to (35), adaptive laws are designed as follows:

(36) $$\left\{ \begin{array}{l} {\dot{\hat{W}}}_{2}=-\Gamma_{2}S_{2}k_{v_{2}}-m_{2}{\hat{W}}_{2}\\ {\dot{\hat{W}}}_{41}=-\Gamma_{41}S_{41}k_{v_{4}}-m_{41}{\hat{W}}_{41}\\ {\dot{\hat{W}}}_{42}=-\Gamma_{42}S_{42}k_{v_{4}}-m_{42}{\hat{W}}_{42}. \end{array}\right.$$

Taking the time derivative of $z_{s}$, and combining with the virtual control signals, we then have:

(37) $$\begin{array}{ll} {\dot{z}}_{s} & ={\dot{z}}_{42}-{\dot{z}}_{41}\\ & =a_{0}[\hbar_{12}x_{52}-\hbar_{22}(x_{32}-x_{1})+\Phi_{42}-\Phi_{52}-\hbar_{32}x_{42}-\hbar_{11}x_{51}+\\ & \hbar_{21}(x_{31}-x_{1})-\Phi_{41}+\Phi_{51}+\hbar_{31}x_{41}]\\ & =a_{0}\hbar_{12}x_{52}-x_{52,c}-a_{0}\hbar_{11}x_{51}+x_{51,c}+(x_{52,c}-\alpha_{42}-x_{51,c}+\alpha_{41})-\\ & \alpha_{41}+\alpha_{42}+a_{0}(\hbar_{21}x_{31}-\hbar_{21}x_{1}+\hbar_{31}x_{41}-\hbar_{22}x_{32}+\hbar_{22}x_{1}-\hbar_{32}x_{42})+\\ & a_{0}(\Phi_{42}-\Phi_{41})+a_{0}(\Phi_{51}-\Phi_{52})\\ & =z_{T}+(x_{52,c}-\alpha_{42}-x_{51,c}+\alpha_{41})-2k_{S}z_{s}+({\tilde{W}}_{42}^{T}S_{42}-\varsigma_{42})+\\ & \left(-{\tilde{W}}_{41}^{T}S_{41}+\varsigma_{41})+a_{0}(\Phi_{51}-\Phi_{52}\right)\\ & \leq z_{T}-2k_{S}z_{s}+\beta_{0}+\vartheta +\varepsilon_{0}+H_{3}\\ & =z_{T}-2k_{S}z_{s}+\lambda , \end{array}$$

in which $a_{0}(\Phi_{51}-\Phi_{52})<H_{3}$, $\beta_{0}+\vartheta +\varepsilon_{0}+H_{3}=\lambda$. $\beta_{0},\vartheta ,\varepsilon_{0},H_{3}$ and λ are all positive parameters.

**Remark** **6.** *From Lemma 3, we know*$\left|x_{52,c}-\alpha_{42}-x_{51,c}+\alpha_{41}\right|\leq \beta_{0}$*if appropriate filtering parameters are selected. The speed of the two motors is required to be synchronized, that is, the speed difference between them is almost zero. The only different variable of the two approximation parts is the speed, so*${\tilde{W}}_{42}^{T}S_{42}-{\tilde{W}}_{41}^{T}S_{41}$*is bounded, and denoted as*$({\tilde{W}}_{42}^{T}S_{42}-{\tilde{W}}_{41}^{T}S_{41})\leq \vartheta$. *From Assumption 1, we can easily know that*$(\varsigma_{41}-\varsigma_{42})\leq \varepsilon_{0}$.

Similarly, the ${\dot{z}}_{T}$ is calculated:

(38) $$\begin{array}{ll} {\dot{z}}_{T} & ={\dot{z}}_{52}-{\dot{z}}_{51}\\ & =a_{0}\hbar_{12}{\dot{x}}_{52}-{\dot{x}}_{52,c}-a_{0}\hbar_{11}{\dot{x}}_{51}+{\dot{x}}_{51,c}\\ & =a_{0}\hbar_{12}(-\hbar_{42}x_{52}-\hbar_{52}x_{42}+\hbar_{62}U_{2})-{\dot{x}}_{52,c}-a_{0}\hbar_{11}(-\hbar_{41}x_{51}\\ & -\hbar_{51}x_{41}+\hbar_{61}U_{1})+{\dot{x}}_{51,c}\\ & =-(k_{5}+2a_{0}k_{T})z_{T}-z_{s}. \end{array}$$

From (37) and (38), we can obtain:

(39) $$z_{s}{\dot{z}}_{s}+z_{T}{\dot{z}}_{T}=-2k_{S}z_{s}^{2}-(k_{5}+2a_{0}k_{T})z_{T}^{2}+\lambda z_{s}\leq -(2k_{S}-1)z_{s}^{2}-(k_{5}+2a_{0}k_{T})z_{T}^{2}+\frac{1}{4}\lambda^{2}.$$

Combining the Equations (35), (36), and (39), we conclude:

(40) $$\begin{array}{ll} \dot{V} & ={\dot{V}}_{5}+{\tilde{W}}_{2}{}^{T}\Gamma_{2}^{-1}{\dot{\hat{W}}}_{2}+{\tilde{W}}_{41}{}^{T}\Gamma_{41}^{-1}{\dot{\hat{W}}}_{41}+{\tilde{W}}_{42}{}^{T}\Gamma_{42}^{-1}{\dot{\hat{W}}}_{42}+z_{s}{\dot{z}}_{s}+z_{T}{\dot{z}}_{T}\\ & \leq -\sum_{i=1}^{5}k_{v_{i}}k_{i}v_{i}-m_{2}{\tilde{W}}_{2}^{T}\Gamma_{2}^{-1}{\hat{W}}_{2}-m_{41}{\tilde{W}}_{41}^{T}\Gamma_{41}^{-1}{\hat{W}}_{41}-m_{42}{\tilde{W}}_{42}^{T}\Gamma_{42}^{-1}{\hat{W}}_{42}\\ & -(2k_{S}-1)z_{s}^{2}-(k_{5}+2a_{0}k_{T})z_{T}^{2}+\frac{1}{4}\lambda^{2}+\frac{1}{2}\varepsilon_{2}^{2}+\frac{1}{2}H_{2}^{2}+\frac{1}{2}\varepsilon_{41}^{2}+\\ & \frac{1}{2}H_{41}^{2}+\frac{1}{2}\varepsilon_{42}^{2}+\frac{1}{2}H_{42}^{2}\\ & \leq -\sum_{i=1}^{5}k_{v_{i}}k_{i}v_{i}-\frac{m_{2}}{2}{\tilde{W}}_{2}{}^{T}\Gamma_{2}^{-1}{\tilde{W}}_{2}-\frac{m_{41}}{2}{\tilde{W}}_{41}{}^{T}\Gamma_{41}^{-1}{\tilde{W}}_{41}-\frac{m_{42}}{2}{\tilde{W}}_{42}{}^{T}\Gamma_{42}^{-1}{\tilde{W}}_{42}\\ & -(2k_{S}-1)z_{s}^{2}-(k_{5}+2a_{0}k_{T})z_{T}^{2}+\frac{m_{2}}{2}W_{2}^{*}{}^{T}\Gamma_{2}^{-1}W_{2}^{*}+\frac{m_{41}}{2}W_{41}^{*}{}^{T}\Gamma_{41}^{-1}W_{41}^{*}+\\ & \frac{m_{42}}{2}W_{42}^{*}{}^{T}\Gamma_{42}^{-1}W_{42}^{*}+\frac{1}{4}\lambda^{2}+\frac{1}{2}\varepsilon_{2}^{2}+\frac{1}{2}H_{2}^{2}+\frac{1}{2}\varepsilon_{41}^{2}+\frac{1}{2}H_{41}^{2}+\frac{1}{2}\varepsilon_{42}^{2}+\frac{1}{2}H_{42}^{2}. \end{array}$$

According to Lemma 1, we obtain:

(41) $$\begin{array}{ll} \dot{V} & \leq -\sum_{i=1}^{5}k_{i}\log(\frac{k_{b_{i}}^{2}(t)}{k_{b_{i}}^{2}(t)-v_{i}^{2}})-\frac{m_{2}}{2}{\tilde{W}}_{2}{}^{T}\Gamma_{2}^{-1}{\tilde{W}}_{2}-\frac{m_{41}}{2}{\tilde{W}}_{41}{}^{T}\Gamma_{41}^{-1}{\tilde{W}}_{41}-\\ & \frac{m_{42}}{2}{\tilde{W}}_{42}{}^{T}\Gamma_{42}^{-1}{\tilde{W}}_{42}-(2k_{S}-1)z_{s}^{2}-(k_{5}+2a_{0}k_{T})z_{T}^{2}+\frac{m_{2}}{2}W_{2}^{*}{}^{T}\Gamma_{2}^{-1}W_{2}^{*}\\ & +\frac{m_{41}}{2}W_{41}^{*}{}^{T}\Gamma_{41}^{-1}W_{41}^{*}+\frac{m_{42}}{2}W_{42}^{*}{}^{T}\Gamma_{42}^{-1}W_{42}^{*}+\frac{1}{4}\lambda^{2}+\frac{1}{2}\varepsilon_{2}^{2}+\frac{1}{2}H_{2}^{2}+\frac{1}{2}\varepsilon_{41}^{2}+\\ & \frac{1}{2}H_{41}^{2}+\frac{1}{2}\varepsilon_{42}^{2}+\frac{1}{2}H_{42}^{2}\\ & \leq -q_{0}V+p_{0}, \end{array}$$

where $q_{0}=\min\{2k_{1},2k_{2},2k_{3},2k_{4},2k_{5},m_{2},m_{41},m_{42},2(2k_{S}-1),2(k_{5}+2a_{0}k_{T})\},$ $\begin{array}{l} p_{0}=\frac{m_{2}}{2}W_{2}^{*}{}^{T}\Gamma_{2}^{-1}W_{2}^{*}+\frac{m_{41}}{2}W_{41}^{*}{}^{T}\Gamma_{41}^{-1}W_{41}^{*}+\frac{m_{42}}{2}W_{42}^{*}{}^{T}\Gamma_{42}^{-1}W_{42}^{*}+\frac{1}{4}\lambda^{2}+\frac{1}{2}\varepsilon_{2}^{2}+\frac{1}{2}H_{2}^{2}+\frac{1}{2}\varepsilon_{41}^{2}+\\ \frac{1}{2}H_{41}^{2}+\frac{1}{2}\varepsilon_{42}^{2}+\frac{1}{2}H_{42}^{2}. \end{array}$

From (41), we can obtain:

(42) $$V\leq (V(t_{0})-p_{0}/q_{0})e^{-q_{0}(t-t_{0})}+p_{0}/q_{0}\leq V(t_{0})+p_{0}/q_{0},\forall t\geq t_{0},\;\;\;\;\;$$

further

(43) $$\log(\frac{k_{b_{i}}^{2}(t)}{k_{b_{i}}^{2}(t)-v_{i}^{2}})\leq 2(V(t_{0})-p_{0}/q_{0})e^{-q_{0}(t-t_{0})}+2p_{0}/q_{0}.$$

Take the natural logarithm for (43) and we have $\frac{k_{b_{i}}^{2}(t)}{k_{b_{i}}^{2}(t)-v_{i}^{2}}\leq e^{2(V(t_{0})-p_{0}/q_{0})e^{-q_{0}(t-t_{0})}+2p_{0}/q_{0}}$. Thus, we have $\left|v_{i}\right|\leq k_{b_{i}}\sqrt{1-e^{-2(V(t_{0})-p_{0}/q_{0})e^{-q_{0}(t-t_{0})}-2p_{0}/q_{0}}}$.

When t→∞, we get:

(44) $$\left|v_{i}\right|\leq k_{b_{i}}\sqrt{1-e^{-2p_{0}/q_{0}}}.$$

A Lyapunov function was designed for the compensation signal system as:

(45) $$V_{0}=\frac{1}{2}\xi_{1}^{2}+\frac{1}{2}\xi_{2}^{2}+\frac{1}{2}\xi_{3}^{2}+\frac{1}{2}\xi_{4}^{2}+\frac{1}{2}\xi_{5}^{2}.$$

It can be obtained by the time derivative of $V_{0}$:

(46) $$\begin{array}{ll} {\dot{V}}_{0} & =\xi_{1}{\dot{\xi }}_{1}+\xi_{2}{\dot{\xi }}_{2}+\xi_{3}{\dot{\xi }}_{3}+\xi_{4}{\dot{\xi }}_{4}+\xi_{5}{\dot{\xi }}_{5}\\ & =-k_{1}\xi_{1}^{2}-k_{2}\xi_{2}^{2}-k_{3}\xi_{3}^{2}-k_{4}\xi_{4}^{2}-k_{5}\xi_{5}^{2}+\xi_{1}\xi_{2}+\xi_{2}\xi_{3}+\xi_{3}\xi_{4}+\xi_{4}\xi_{5}\\ & +\xi_{1}(x_{2,c}-\alpha_{1})+\xi_{2}(x_{3,c}-\alpha_{2})+\xi_{3}(x_{4,c}-\alpha_{3})+\xi_{4}(x_{51,c}-\alpha_{41}+x_{52,c}-\alpha_{42}). \end{array}$$

According to the Lemma 3, we know that $\left|x_{2,c}-\alpha_{1}\right|\leq \beta_{1},\left|x_{3,c}-\alpha_{2}\right|\leq \beta_{2},\left|x_{4,c}-\alpha_{3}\right|\leq \beta_{3}$ and $\left|x_{51,c}-\alpha_{41}+x_{52,c}-\alpha_{42}\right|\leq \beta_{4}$.

Therefore, we have:

(47) $$\begin{array}{l} {\dot{V}}_{0}\leq -(k_{1}-1)\xi_{1}^{2}-(k_{2}-\frac{3}{2})\xi_{2}^{2}-(k_{3}-\frac{3}{2})\xi_{3}^{2}-(k_{4}-\frac{3}{2})\xi_{4}^{2}-(k_{5}-\frac{1}{2})\xi_{5}^{2}+\\ \frac{1}{2}\beta_{1}^{2}+\frac{1}{2}\beta_{2}^{2}+\frac{1}{2}\beta_{3}^{2}+\frac{1}{2}\beta_{4}^{2}\\ \leq -n_{0}V_{0}+m_{0}, \end{array}$$

where $n_{0}=\min\{2(k_{1}-1),2k_{2}-3,2k_{3}-3,2k_{4}-3,2k_{5}-1\},m_{0}=\frac{1}{2}\beta_{1}^{2}+\frac{1}{2}\beta_{2}^{2}+\frac{1}{2}\beta_{3}^{2}+\frac{1}{2}\beta_{4}^{2}$, $\beta_{1},\beta_{2},\beta_{3},\beta_{4}$ are positive parameters.

According to (47), when t→∞, we have:

(48) $$\left|\xi_{i}\right|\leq \sqrt{2m_{0}/n_{0}}.$$

From the error systems designed, we can obtain $\left|z_{1}\right|\leq \left|v_{1}\right|+\left|\xi_{1}\right|\leq k_{b_{1}}\sqrt{1-e^{-2p_{0}/q_{0}}}+\sqrt{2m_{0}/n_{0}}$ by combining (44) and (48). It denotes that the tracking error tends to a small neighborhood of the origin if the control parameters are selected properly. In the actual applications of the dual-motor servo system, there exists a positive constant Y, making $\left|y_{d}\right|<Y<k_{c_{1}}$, so $\left|x_{1}\right|\leq \left|z_{1}\right|+\left|y_{d}\right|\leq k_{b_{1}}+\sqrt{2m_{0}/n_{0}}+Y\leq k_{c_{1}}$. We know the virtual control signal $\alpha_{1}$ is bounded, satisfying $\left|\alpha_{1}\right|<\sigma_{1}$. In addition, $\left|x_{2,c}-\alpha_{1}\right|\leq \beta_{1}$, so $\left|x_{2,c}\right|\leq \sigma_{1}+\beta_{1}\leq \tau_{1}$. Ultimately, we have $\left|x_{2}\right|\leq \left|z_{2}\right|+\left|x_{2,c}\right|<k_{b_{2}}+\sqrt{2m_{0}/n_{0}}+\tau_{1}<k_{c_{2}}$. Similarly, we also get $\left|x_{i}\right|\leq k_{c_{i}},i=3,4,5$. □

## 5. Simulation

In this section, the simulation results show better control performance of adaptive NNs based on CFB considering full-state constraints. The application of this method can achieve good control performance for tracking the desired reference signal and reducing the synchronization error in dual-motor servo systems with partial asymmetric dead-zone. The parameters of motors are given as follows

$$\left\{ \begin{array}{l} J_{m1}=4\times 10^{-3}kg\times m^{2}\\ K_{e1}=0.76V/rad\\ K_{t1}=1.1N\cdot m/A\\ R_{1}=2.5\Omega \\ L_{1}=5\times 10^{-2}H, \end{array}\right.\left\{ \begin{array}{l} J_{m2}=8\times 10^{-3}kg\times m^{2}\\ K_{e2}=0.51V/rad\\ K_{t2}=0.9N\cdot m/A\\ R_{2}=3\Omega \\ L_{2}=4\times 10^{-2}H. \end{array}\right.$$

The inertia of the load is defined as $J_{L}=2(J_{m1}+J_{m2})$. The control parameters are chosen in the desired range to guarantee the boundedness of signals, constraints of the state, and the stability of the closed-loop system. Thus, the selected filter parameters and control parameters are: $\omega_{n}=11,000,\zeta =1,k_{1}=1000,k_{2}=80,k_{3}=20,k_{4}=10,k_{5}=10,k_{S}=9000,k_{T}=1.$ The designed parameters in adaptive laws are as follows:

$$\begin{array}{l} m_{2}=1\times 10^{-3},m_{41}=1\times 10^{-3},m_{42}=1\times 10^{-3},\\ \Gamma_{2}=diag[8\times 10^{2},8\times 10^{2},8\times 10^{2},8\times 10^{2},8\times 10^{2}],\\ \Gamma_{41}=diag[6\times 10^{6},6\times 10^{6},6\times 10^{6},6\times 10^{6},6\times 10^{6}],\\ \Gamma_{42}=diag[6\times 10^{6},6\times 10^{6},6\times 10^{6},6\times 10^{6},6\times 10^{6}]. \end{array}$$

In the dead-zone model and time-varying bounded functions, $k_{b_{i}}$, the parameters selected were:

$$\begin{array}{l} r=10,k=4,\partial_{r}=0.0001,\partial_{l}=0.0002,\\ \iota_{1}=0.2,\iota_{2}=0.3,\iota_{3}=0.25,\iota_{4}=0.2,\iota_{5}=0.24,\\ \psi_{1}=0.002,\psi_{2}=0.001,\psi_{3}=0.002,\psi_{4}=0.001,\psi_{5}=0.002,\\ \gamma_{1}=20,\gamma_{2}=25,\gamma_{3}=20,\gamma_{4}=25,\gamma_{5}=20. \end{array}$$

The membership functions were designed as:

$$\begin{array}{l} s_{1}(x_{i})=\exp(-{\left(x_{i}-8\right)}^{T}(x_{i}-8)/6),\\ s_{2}(x_{i})=\exp(-{\left(x_{i}-4\right)}^{T}(x_{i}-4)/6),\\ s_{3}(x_{i})=\exp(-{\left(x_{i}-0\right)}^{T}(x_{i}-0)/6),\\ s_{4}(x_{i})=\exp(-{\left(x_{i}+4\right)}^{T}(x_{i}+4)/6),\\ s_{5}(x_{i})=\exp(-{\left(x_{i}+8\right)}^{T}(x_{i}+8)/6). \end{array}$$

**Figure 3:**
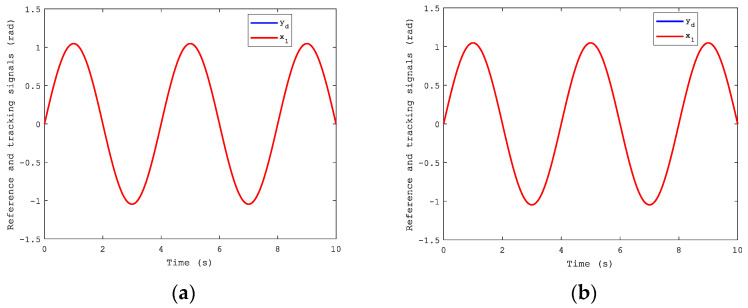
Position tracking performance: (**a**) with constraints; (**b**) without constraints.

**Figure 4:**
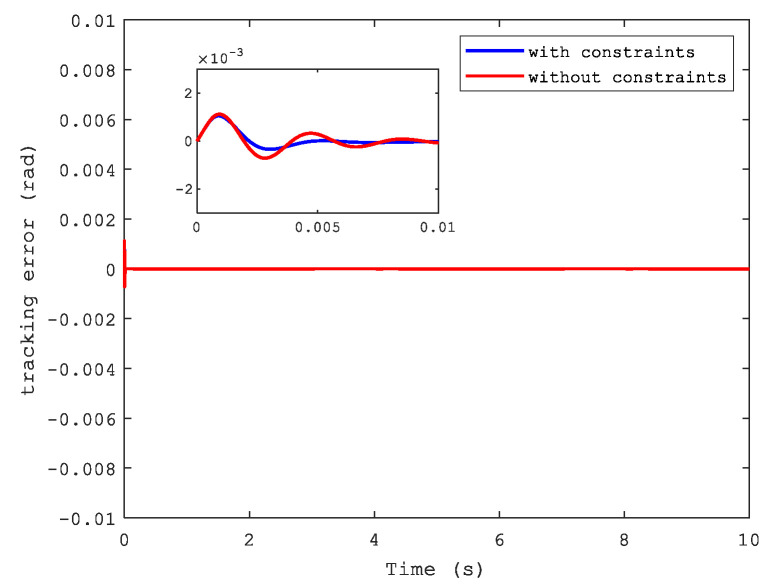
Tracking error.

**Figure 5:**
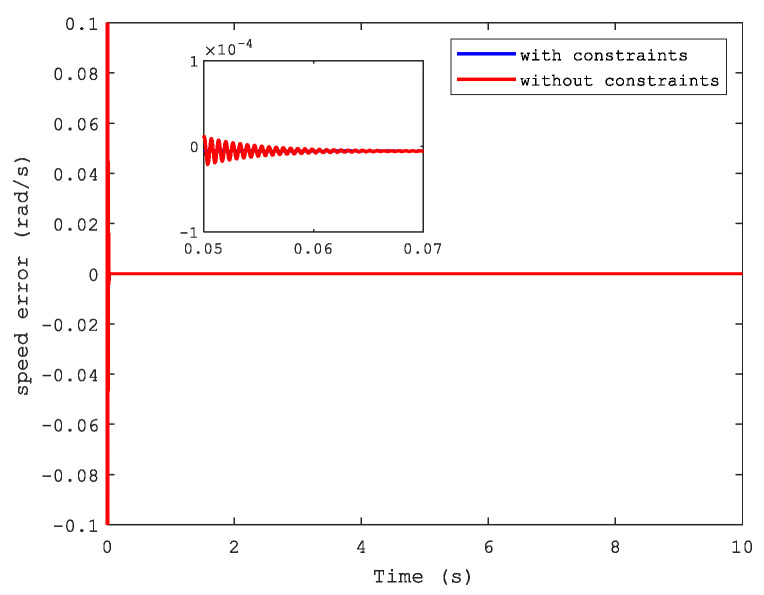
Speed error.

**Figure 6:**
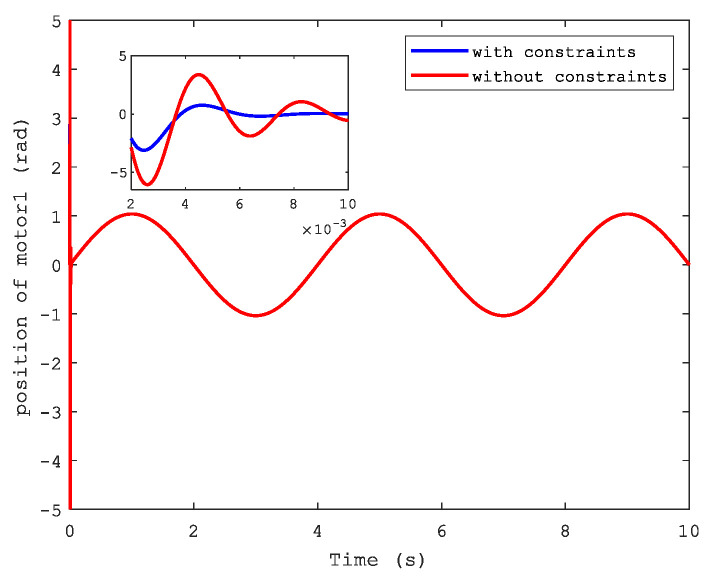
Position of motor 1.

**Figure 7:**
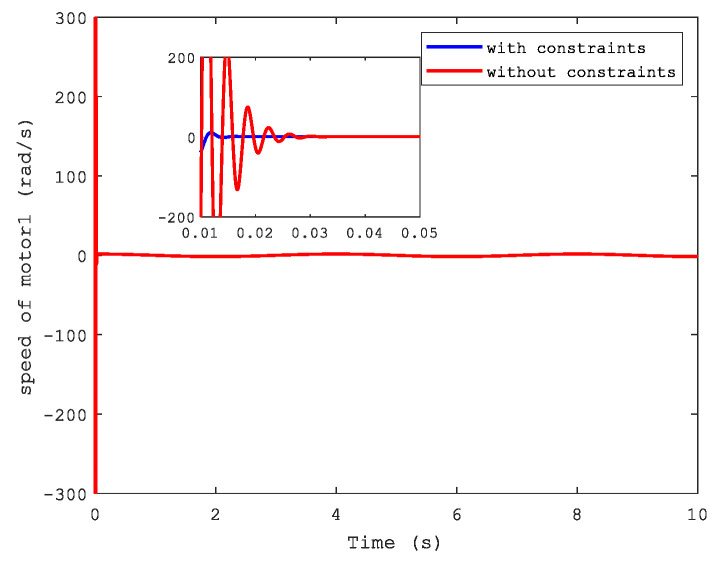
Speed of motor 1.

**Figure 8:**
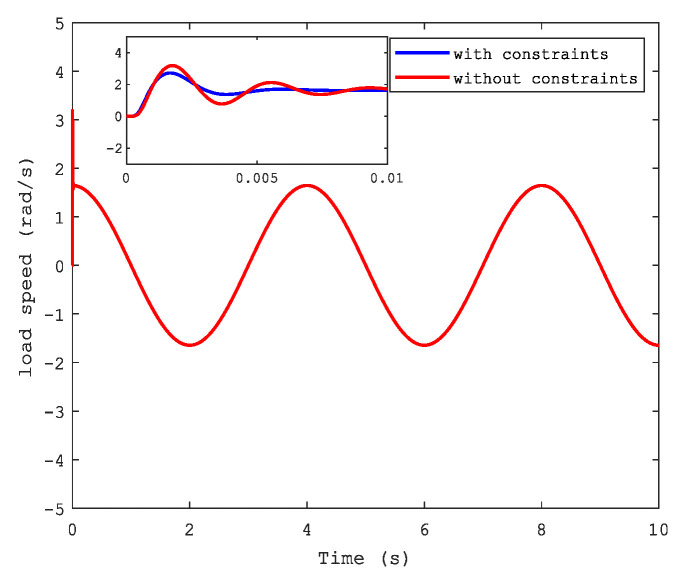
Load speed.

**Figure 9:**
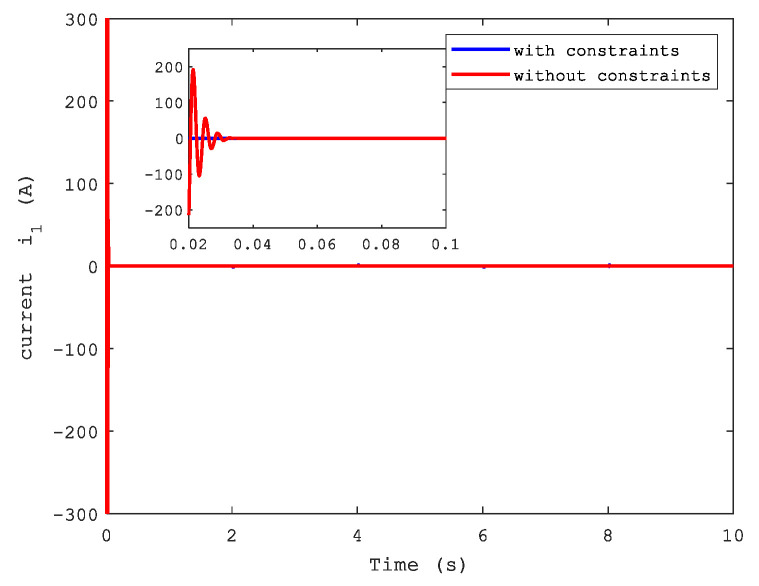
Current $i_{1}$.

**Figure 10:**
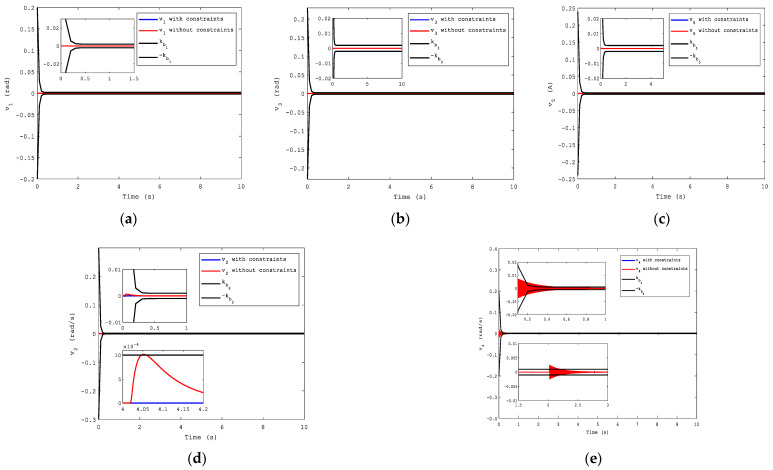
Compensated tracking errors: (**a**) for the first subsystem; (**b**) for the third subsystem; (**c**) for the fifth subsystem; (**d**) for the second subsystem; (**e**) for the fourth subsystem.

The expected tracking signal was a sinusoidal signal, $y_{d}=\frac{\pi }{3}\sin(\frac{\pi }{2}t)$. To show the effectiveness of the proposed algorithm, NNs based on CFB without considering state constraints were applied to compare control performances with it. We can see the advantages of the CFB with state constraints in Figures 3–10.

Figure 3 shows the position of the tracking performance of the dual-motor servo system under the sinusoidal reference signal that contains CFB with and without constraints. From it, a fairly good tracking performance was obtained, and the effectiveness of our proposed method was proved.

The tracking error and synchronization error of the system are shown by Figure 4 and Figure 5. Apparently, it is easy to see that the tracking performance was superior when we reflect on the state constraints of the dual-motor system as well as the synchronization error. Therefore, it is necessary to consider this situation in a closed-loop system according to practical applications.

**Remark** **7.** *Since the position and speed curves of motor 2 differ only one error from that of motor 1, and we have known that the error between them was very small from Figure 5, at this point, only the curves of motor 1 are presented and that of motor 2 are omitted*.

Figure 6 and Figure 7 show the position and speed of motor 1 separately under CFB with and without constraints. A better performance can be seen in the figures with state constraints. The trajectories of load speed and the current, $i_{1}$, are illustrated respectively in Figure 8 and Figure 9.

**Remark** **8.** *The current of motor 1 was similar to that of motor 2, so the latter is not presented here*.

The compensated tracking errors are shown in Figure 10. Evidently, when considering the state constraints, the system showed superior dynamic performance, and all the compensated tracking errors did not go beyond the boundaries. However, when this case was not considered in the system, the second and fourth compensated tracking errors exceed the time-varying boundaries as shown in Figure 10d,e, which caused the violation of the state constraints in the system.

**Remark** **9.** *In practical applications, if this situation is not taken into consideration, it is probable that instability and even greater losses are caused in the entire system. Thus, it makes sense to do that. In brief, the CFB with full-state constraints via T-BLF in this paper can ensure that the constraints are not transgressed*.

## 6. Conclusions

In this paper, the CFB considering full-state constraints for the dual-motor servo system with partial asymmetric dead-zone was investigated via T-BLF. The proposed T-BLF satisfied the requirement of time-varying constraints in practice occasions compared with other existing constrained schemes. The CFB was applied to the dual-motor system, avoiding the complex computational explosion problems. In addition, an error compensation mechanism was introduced that could effectively reduce the filtering errors of the system. Based on CFB, the adaptive NNs could well approximate the nonlinear parts of the dead-zone model and reduce the adverse effects of this part on the system. Through use of the control schemes, all signals were uniformly ultimately bounded, and the state constraints were not violated in the closed-loop system. The tracking error and synchronization error converged to a small neighborhood of the origin in arbitrary precision. To a great extent, the control performance of the dual-motor servo system improved.

## Data Availability

Not applicable.

## References

[1] Chen W.D., Yung K.L., Cheng K.W. (2006). A learning scheme for low-speed precision tracking control of hybrid stepping motors. IEEE/ASME Trans. Mechatron..

[2] Jin F.J., Huang M.S., Chen S.G. (2019). Intelligent maximum torque per ampere tracking control of synchronous reluctance motor using recurrent Legendre fuzzy neural network. IEEE Trans. Power Electron..

[3] Thomas J., Hansson A. (2013). Speed tracking of a linear induction motor-enumerative nonlinear model predictive control. IEEE Trans. Control Syst. Technol..

[4] Lei W., Li C., Chen M.Z.Q. (2019). Robust adaptive tracking control for quadrotors by combining PI and self-tuning regulator. IEEE Trans. Control Syst. Technol..

[5] Kim S.K., Lee K.G., Lee K.B. (2016). Singularity-free adaptive speed tracking control for uncertain permanent magnet synchronous motor. IEEE Trans. Power Electron..

[6] Zhao H.B., Wang C.G., Song Y. (2016). All-coeffificient adaptive control of dual-motor driving servo system. J. Jinggangshan Univ. Nat. Sci..

[7] Zhao H.B., Zhou X.H. (2011). Backsteppig adaptive control of dual-motor driving servo system. Control Theory & Appl..

[8] Zeng T.Y., Ren X.M., Zhang Y. (2020). Fixed-time sliding mode control and high-gain nonlinearity compensation for dual-motor driving system. IEEE Trans. Ind. Inform..

[9] Hu M.H., Zeng J.F., Xu S.Z., Fu C.Y., Qin D.T. (2015). Efficiency study of a dual-motor coupling EV powertrain. IEEE Trans. Veh. Technol..

[10] Wu L., Wang L.H., Zhang C.Y., Shi H.Y. (2019). Dynamic characteristics analysis and dual motor synchronous control of hydraulic lifting system for large cranes. J. Eng..

[11] Zhang G.B., Liu J.P., Liu Z.J., Yu J.P., Ma Y.M. (2020). Adaptive fuzzy discrete-time fault-tolerant control for permanent magnet synchronous motors based on dynamic surface technology. Neurocomputing.

[12] Yu J.P., Shi P., Dong W.J., Chen B., Lin C. (2015). Neural network-based adaptive dynamic surface control for permanent magnet synchronous motors. IEEE Trans. Neural Netw. Learn. Syst..

[13] Yu J.P., Ma Y.M., Yu H.S., Lin C. (2017). Adaptive fuzzy dynamic surface control for induction motors with iron losses in electric vehicle drive systems via backstepping. Inf. Sci..

[14] Yu J.P., Zhao L., Yu H.S., Lin C., Dong W.J. (2018). Fuzzy finite-time command filtered control of nonlinear systems with input saturation. IEEE Trans. Cybern..

[15] Han Y., Yu J.P., Zhao L., Yu H.S., Lin C. (2018). Finite-time adaptive fuzzy control for induction motors with input saturation based on command filtering. IET Control Theory Appl..

[16] Luo R.C., Deng Y.P., Xie Y.L. (2020). Neural network backstepping controller design for uncertain permanent magnet synchronous motor drive chaotic systems via command filter. Front. Phys..

[17] Yu J.P., Shi P., Dong W.J., Lin C. (2018). Adaptive fuzzy control of nonlinear systems with unknown dead zones based on command filtering. IEEE Trans. Fuzzy Syst..

[18] Sun L., Huo W., Jiao Z.X. (2017). Adaptive backstepping control of spacecraft rendezvous and proximity operations with input saturation and full-state constraint. IEEE Trans. Ind. Electron..

[19] He W., Chen Y.H., Yin Z. (2016). Adaptive neural network control of an uncertain robot with full-state constraints. IEEE Trans. Cybern..

[20] Yuan Y., Wang Z., Guo L., Liu H.P. (2020). Barrier Lyapunov functions-based adaptive fault tolerant control for flexible hypersonic flight vehicles with full state constraints. IEEE Trans. Syst. Man, Cybern. Syst..

[21] Yang C.G., Huang D.Y., He W., Cheng L. (2020). Neural control of robot manipulators with trajectory tracking constraints and input saturation. IEEE Trans. Neural Netw. Learn. Syst..

[22] Zhang S., Dong Y.T., Ouyang Y.C., Yin Z., Peng K.X. (2018). Adaptive neural control for robotic manipulators with output constraints and uncertainties. IEEE Trans. Neural Netw. Learn. Syst..

[23] He W., David A.O., Yin Z., Sun C.Y. (2016). Neural network control of a robotic manipulator with input deadzone and output constraint. IEEE Trans. Syst. Man, Cybern. Syst..

[24] Zhao Z., He W., Ge S.S. (2014). Adaptive neural network control of a fully actuated marine surface vessel with multiple output constraints. IEEE Trans. Control Syst. Technol..

[25] Ouyang Y.C., Dong L., Xue L., Sun C.Y. (2019). Adaptive control based on neural networks for an uncertain 2-DOF helicopter system with input deadzone and output constraints. IEEE/CAA J. Autom. Sinica.

[26] Kong L.H., He W., Yang C.G., Li Z.J., Sun C.Y. (2019). Adaptive fuzzy control for coordinated multiple robots with constraint using impedance learning. IEEE Trans. Cybern..

[27] Liu Y.J., Lu S.M., Li D.J., Tong S.C. (2017). Adaptive controller design-based ABLF for a class of nonlinear time-varying state constraint systems. IEEE Trans. Syst. Man, Cybern. Syst..

[28] Yang C.G., Jiang Y.M., Na J., Li Z.J., Cheng L., Su C.-Y. (2019). Finite-time convergence adaptive fuzzy control for dual-arm robot with unknown kinematics and dynamics. IEEE Trans. Fuzzy Syst..

[29] Du R.H., Wu Y.F., Chen W., Chen Q.W. (2013). Adaptive fuzzy control for the servo system with LuGre friction. Control Decis..

[30] Yang C.G., Peng G.Z., Li Y.A., Cui R.X., Cheng L., Li Z.J. (2018). Neural networks enhanced adaptive admittance control of optimized robot-environment interaction. IEEE Trans. Cybern..

[31] Wang S.B., Chen Q., Ren X.M., Yu H.S. (2020). Neural network-based adaptive funnel sliding mode control for servo mechanisms with friction compensation. Neurocomputing.

[32] Yang Y.N., Yan Y. (2018). Backstepping sliding mode control for uncertain strict-feedback nonlinear systems using neural-network-based adaptive gain scheduling. J. Syst. Eng. Electron..

[33] Huang H.H., Zhang T., Yang C.G., Chen C.L.P. (2020). Motor learning and generalization using broad learning adaptive neural control. IEEE Trans. Ind. Electron..

[34] Peng G.Z., Chen C.L.P., He W., Yang C.G. (2021). Neural-learning-based force sensorless admittance control for robots with input deadzone. IEEE Trans. Ind. Electron..

[35] Xu Z.H., Li S., Zhou X.F., Zhou S.B., Cheng T.B., Guan Y.S. (2021). Dynamic neural networks for motion-force control of redundant manipulators: An optimization perspective. IEEE Trans. Ind. Electron..

[36] Yang C.G., Chen C.Z., He W., Cui R.X., Li Z.J. (2019). Robot learning system based on adaptive neural control and dynamic movement primitives. IEEE Trans. Neural Netw. Learn. Syst..

[37] Xu Z.H., Zhou X.F., Wu H.M., Li X.X., Li S. (2021). Motion planning of manipulators for simultaneous obstacle avoidance and target tracking: An RNN approach with guaranteed performance. IEEE Trans. Ind. Electron..

[38] Cui E.C., Jing Y.W., Gao X.T. (2020). Full state constraints control of switched complex networks based on time-varying barrier Lyapunov functions. IET Control Theory Appl..

[39] Cai M.J., Xiang Z.R., Guo J. (2016). Adaptive finite-time fault-tolerant consensus protocols for multiple mechanical systems. J. Franklin Inst..

[40] Wang B.F., Iwasaki M., Yu J.P. (2021). Command filtered adaptive backstepping control for dual-motor servo systems with torque disturbance and uncertainties. IEEE Trans. Ind. Electron..

